# An Electronic Clinical Decision-Making Tool for Patients with Suspected Colorectal Cancer—Preliminary Evaluation in Patients Presenting with Rectal Bleeding

**DOI:** 10.1093/jcag/gwz013

**Published:** 2019-05-22

**Authors:** Nauzer Forbes, Mohan Cooray, Michael Hackett, Nishwa Shah, Yuhong Yuan, Pavel Antiperovitch, Tracey Corner, David Chan, Michael Mills, David Armstrong, Ted Xenodemetropoulos

**Affiliations:** 1 Division of Gastroenterology, Department of Medicine, University of Calgary, Calgary, Alberta, Canada; 2 Division of Gastroenterology, Department of Medicine, McMaster University, Hamilton, Ontario, Canada; 3 Faculty of Health Sciences, McMaster University, Hamilton, Ontario, Canada; 4 Farncombe Family Digestive Research Institute, Hamilton, Ontario, Canada; 5 Department of Medicine, Western University, London, Ontario, Canada; 6 Hamilton Health Sciences, Hamilton, Ontario, Canada; 7 Faculty of Health Sciences, Department of Family Medicine, McMaster University, Hamilton, Ontario, Canada; 8 Halton McMaster Family Health Centre, Burlington, Ontario, Canada

**Keywords:** Colonoscopy, Colorectal neoplasms, Decision making

## Abstract

**Background and Objectives:**

The *CarePath-CRC* electronic clinical decision-making application was designed to assist physicians with evaluation of patients with suspected colorectal cancer (CRC). The physician completes an interactive checklist of evidence-based clinical parameters, and a recommended referral urgency is generated based on the post-test probability of CRC. This study aimed toward validation of the tool in symptomatic patients presenting with rectal bleeding.

**Methods:**

The medical records of a sample of patients with histologically confirmed CRC from 2010 to 2014 were reviewed. The *CarePath-CRC* tool was applied retrospectively to all patients who initially presented with rectal bleeding, to determine its sensitivity for detecting CRC in this population. A generated recommendation of ‘immediate referral’ (referral ≤24 hours, expected endoscopy ≤2 weeks) or ‘urgent referral’ (expected consultation and endoscopy ≤4 and ≤8 weeks) was considered a positive test result. An a priori sensitivity of 90% was deemed adequate, based on test characteristics of the tool’s individual clinical criteria.

**Results:**

The tool was applied to 281 patients. A total of 69 (24.6%) and 211 (75.1%) patients met criteria for immediate and urgent referral, respectively. The remaining patient (0.4%) met criteria for ‘possible priority referral’, while none met criteria for ‘no specific action recommended’. This resulted in a calculated sensitivity of 99.6% (95% confidence interval 98.0 to 99.9%).

**Conclusions:**

The *CarePath-CRC* tool is sensitive in the prediction of CRC in patients presenting with rectal bleeding. A prospective cohort study is being designed to allow for acquisition of comprehensive test performance characteristics and full validation of the instrument.

Colorectal cancer (CRC) is the second most prevalent form of malignancy in Canada. The lifetime probability of developing CRC is 1 in 13 for men, and 1 in 16 for women (1). Several systematic programs have been instituted across Canada to screen for CRC; by definition, these screening programs apply to asymptomatic individuals. However, individuals often also present with symptoms, such as rectal bleeding, that may indicate an underlying CRC. Colonoscopy is considered the gold standard investigation, both for screening and in the workup of clinically suspected CRC (2,3). For patients in whom a diagnosis of CRC is suspected, prompt referral for colonoscopy is essential. Delays in this referral process occur because of both patient-related and physician-related factors. Patient-related elements that most often result in delays are lack of patient recognition of the significance of their symptoms and the fear of potential upcoming investigations (4). Physician-related factors include lack of recognition of CRC symptoms in patients, lack of investigation of anemia and lack of performance of a digital rectal examination (5).

Early suspicion is key to prompt referral and diagnosis of CRC in symptomatic patients, but this can be challenging based on clinical parameters alone. Several parameters have been studied, including patient characteristics, clinical signs and symptoms, and laboratory test results. Clinical assessment scores and statistical models have also been developed using these parameters in an effort to assist physicians with accurately identifying patients for whom a high suspicion of CRC exists (6–9). These parameters and scores share some shortcomings, including a high degree of variability in terms of diagnostic utility. In addition, the performance characteristics of most of the available scores favour high sensitivity (with poor specificity) (10–12). Furthermore, the majority of the data used to evaluate the value of these parameters are not from the primary care setting, and none of the data represent the Canadian population.

The *CarePath-CRC* electronic decision-making application was designed to assist family physicians and other primary care providers in determining how to best to manage patients with clinical features suspicious of CRC. It offers guidance by planning a referral pathway using the evidence-based clinical parameters outlined above. Ultimately, the *CarePath* tool is intended to help prioritize the urgency of patient referral for further consultation and colonoscopy. The *CarePath* electronic advisor is intended for application directly at the point of patient care. It comprises two main components: the first is an interactive checklist that leads the physician to consider and report important clinical parameters relevant to the suspicion of CRC; the second instantly tabulates a pre- and post-test probability of CRC based on the information provided, and then delivers recommendations regarding the urgency and appropriate pathway of referral. The clinician then retains the option to save and/or print the report for each patient to whom it is applied. The electronic interface is shown in Figures 1 and 2.

**Figure 1.**
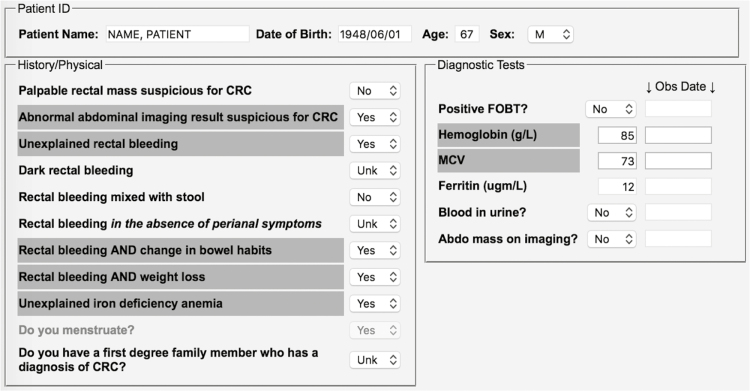
Interactive checklist component of *CarePath-CRC* tool.

**Figure 2.**
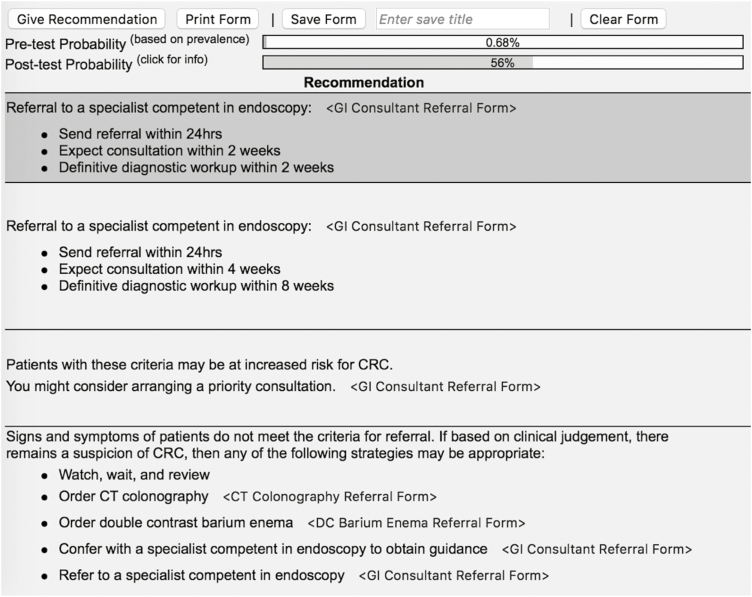
Summary and recommendation component of *CarePath-CRC* tool.

The performance characteristics of previously studied models have been assessed when applied to all patients referred for investigation of CRC, rather than those presenting with rectal bleeding. The *Carepath-CRC* tool distinguishes between different types of rectal bleeding presentations, which the other tools do not do. No tool has specifically assessed the performance of a tool exclusively in patients presenting with rectal bleeding. Furthermore, the *Carepath-CRC* tool is specifically designed for point-of-care use with immediate feedback or referral priority based on available evidence. The *CarePath* tool has not yet been validated in terms of its ability to accurately predict CRC in symptomatic patients with rectal bleeding. As such, in this preliminary evaluation, we aimed to assess the sensitivity of the *CarePath* tool in detecting CRC among patients presenting with rectal bleeding. The investigators wished to ensure an adequate baseline level of sensitivity for the tool prior to proceeding with larger prospective studies.

## METHODS

### Development of the *CarePath* Electronic Tool

The Program in Evidence-based Care (PEBC) is an initiative of Cancer Care Ontario (CCO) supported by the Ontario Ministry of Health and Long-term Care. As part of the PEBC, a working group comprised of physicians and surgeons reviewed the available evidence in 2012 in an attempt to make a series of evidence-based recommendations on the referral of patients with suspected CRC by primary care providers (13). A total of 26 studies reporting on possible signs, symptoms or risk factors for CRC were comprehensively reviewed in terms of quality, and the positive predictive value (PPV) for each parameter was extracted from each study. Following this, the median PPV was calculated for each parameter across all available studies. The working group concluded that the presence of any clinical parameter with a median PPV of 10% or more should prompt an expedited referral for definitive evaluation of suspected CRC.

**Figure 3.**
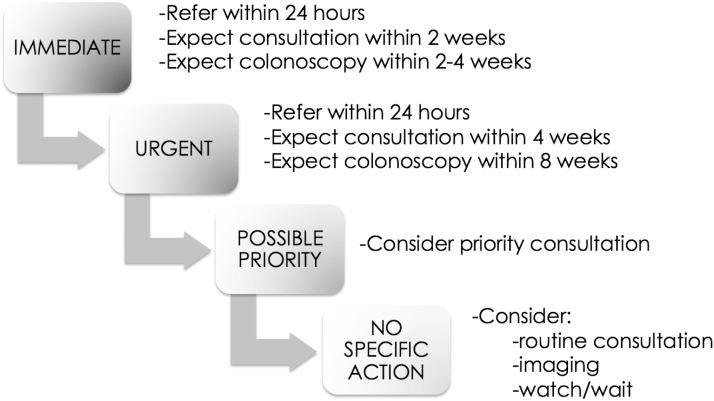
Recommended actions for specific referral urgencies.

Thus, using these criteria (Table 1), a comprehensive electronic platform was designed so that a primary care provider (family physician, emergency physician, nurse practitioner, physician’s assistant, registered nurse) could select the presence or absence of each of these criteria, and others, in real time at the point of care. The tool was designed to give an immediate recommendation to the practitioner based on the inputted data (Figure 3).

**Table 1.** CCO-recommended referral urgencies from clinical and diagnostic parameters

Clinical and diagnostic parameters	Urgency of referral
• Palpable rectal mass suspicious for CRC • Abnormal abdominal imaging result suspicious for CRC	Immediate
• Unexplained rectal bleeding with at least one of: Dark rectal bleeding Rectal bleeding mixed with stool Rectal bleeding in the absence of perianal symptoms Rectal bleeding and change in bowel habits Rectal bleeding and weight loss Unexplained iron deficiency anemia and a hemoglobin of ≤110 g/L for males or ≤100 g/L for nonmenstruating females	Urgent
• Patients age >59 years with any of above signs or symptoms • Male patients with any of above signs or symptoms • A combination of any of above signs or symptoms • Patients with any of above signs or symptoms and a first degree family member with a diagnosis of CRC	Possible Priority
• None of the mentioned features present	No Specific Action

Data taken from ref. (13).

CCO, Cancer Care Ontario; CRC, Colorectal cancer.

### Assigning Referral Priorities

The evidence-based criteria used for the tool to automatically assign referral priorities in real-time are shown in Table 1. A palpable mass or suspicious imaging finding prompts ‘immediate referral’ (referral ≤24 hours, expected endoscopy ≤ 2 weeks), and a history of unexplained iron deficiency anemia, dark rectal bleeding, blood mixed with stools, bleeding in the absence of perianal symptoms, bleeding and changes in bowel habits or bleeding with weight loss prompts ‘urgent referral’ (expected consultation and endoscopy ≤ 4 and ≤ 8 weeks).

### Study Setting and Patients

Hamilton Health Sciences (HHS) is a tertiary care hospital network that serves over two million residents of Hamilton and south central Ontario. The Hamilton CRC Pathway is a system that was implemented to expedite assessment and management of patients with suspected CRC following colonoscopy performed at HHS or at several regional affiliates. All patients within the database were interviewed by the same certified gastroenterology nurse (CGN) using the same series of questions. The CRC Pathway clinical database was reviewed, and all patients over the age of 18 referred to the pathway between 2010 and 2014 with histologically confirmed CRC were included. Ethics approval for the study was obtained through the Hamilton Integrated Research Ethics Board at McMaster University according to institutional practices.

### Study Design and Outcomes

This study represented the first of two planned phases. The first phase, reported here, was a retrospective review of the included patients’ medical records designed to calculate the overall sensitivity of the tool in the detection of CRC in patients presenting with rectal bleeding. If the tool were to meet the predetermined sensitivity threshold, a second prospective evaluation would then be planned to fully describe the tool’s diagnostic properties. Based on the individual clinical parameters comprising the tool, an a priori sensitivity of 90% was deemed sufficient to proceed with the second phase.

The *CarePath-CRC* tool was applied retrospectively to all patients in this database who initially presented with rectal bleeding, to determine its sensitivity for detecting CRC. A generated recommendation of ‘immediate referral’ (referral ≤24 hours, expected endoscopy ≤ 2 weeks) or ‘urgent referral’ (expected consultation and endoscopy ≤ 4 and ≤ 8 weeks) was considered a positive test result. Conversely, a generated recommendation of ‘possible priority referral’ or ‘no specific action’ was considered a negative test result.

### Data Analysis

An initial joint review of randomly selected medical records was performed to ensure feasibility and reproducibility of the study methods. An intra-rater reliability was calculated after having the same reviewer review the same sample 3 months apart, while an inter-rater reliability (Cohen’s kappa) was also calculated between two reviewers reviewing the sample. The sensitivity of the *CarePath* tool was calculated. All data were analyzed using the Statistical Package for the Social Sciences (SPSS) software, version 20.

## RESULTS

The initial joint review of 25 randomly selected medical records yielded an intra-rater reliability of 1.00 and an inter-rater reliability (Cohen’s kappa) of 0.92. A total of 557 CRC Pathway records from 2010 to 2014 were reviewed. Of these, 45 records with insufficient clinical information and 231 records of patients lacking an initial presentation of rectal bleeding were excluded. The *CarePath* tool was applied to the remaining 281 patients, 158 (56.2%) of whom were male. The remaining clinical characteristics of the study patients are shown in Table 2.

**Table 2.** Clinical characteristics of study patients

Characteristic	Mean	Standard deviation	Minimum	Maximum
Age (years)	67.0	13.4	28	95
Hemoglobin (g/L)	123.3	22.3	43.0	174.0
Mean corpuscular volume (fL)	87.6	8.4	49.3	104.4
Ferritin (ng/mL)	139.0	268.9	3.0	1639.0

### Diagnostic Properties

The presence or absence of each variable measured by the tool and the according priority group to which the patients were assigned are shown in Table 3. Of the 281 patients analyzed using the tool, 69 (24.6%) and 211 (75.1%) patients met criteria for immediate and urgent referral, respectively. The remaining patient (0.4%) met criteria for ‘possible priority referral’, while no patients met criteria for ‘no specific action recommended’. This resulted in a calculated sensitivity of 99.6% (95% confidence interval [CI] 98.0 to 99.9%) for the tool. All variables in question were consistently recorded by the same CGN for each patient, except for the presence or absence of perianal symptoms. Given the lack of consistent recording of perianal symptoms, a sensitivity analysis was performed, whereby patients with unclear records were treated as having perianal symptoms and, thus, were assigned to a less urgent referral pathway. Within this analysis, 23 patients were demoted from the ‘urgent referral’ group to the ‘possible priority referral’ group. This resulted in a calculated sensitivity of 91.5% (95% CI 87.4 to 94.3%).

**Table 3.** Relative frequencies of patient variables by assigned priority group

Variable/priority group	Immediate	Urgent	Possible priority	No specific action
Palpable mass (*n*, %)	54 (19.2)	0 (0.0)	0 (0.0)	0 (0.0)
Abnormal imaging on history (*n*, %)	19 (6.8)	0 (0.0)	0 (0.0)	0 (0.0)
History of iron deficiency anemia (*n*, %)	8 (2.8)	41 (14.6)	0 (0.0)	0 (0.0)
Rectal bleeding and weight loss (*n*, %)	25 (8.9)	56 (19.9)	0 (0.0)	0 (0.0)
Rectal bleeding mixed in stool (*n*, %)	28 (10.0)	96 (34.2)	0 (0.0)	0 (0.0)
Rectal bleeding without perianal symptoms (*n*, %)	62 (22.1)	198 (70.5)	0 (0.0)	0 (0.0)
Rectal bleeding and change in bowel habits (*n*, %)	39 (13.9)	128 (45.6)	0 (0.0)	0 (0.0)
Dark rectal bleeding (*n*, %)	5 (1.8)	17 (6.0)	0 (0.0)	0 (0.0)
Rectal bleeding with none of the above (*n*, %)	0 (0.0)	0 (0.0)	1 (0.4)	0 (0.0)

## DISCUSSION

Our study showed that the *CarePath*-*CRC* electronic tool was highly sensitive in the detection of CRC for patients initially presenting with rectal bleeding (99.6%, 95% CI 98.0 to 99.9%). Even after applying a sensitivity analysis that demoted 23 of 281 patients with uncertain perianal symptoms to a lower referral urgency, the calculated sensitivity was 91.5% (95% CI 87.4 to 94.3%). This meets the a priori criteria our group set out for minimum sensitivity required to proceed to the next phase of validation for this tool.

An inherent strength of our study was the use of an established CRC referral pathway database, which allowed for comprehensive centralized review of all medical records in question. All patients assessed within this pathway were done so by the same CGN, a system which contributed a high degree of reliability and reproducibility to the approach and record-keeping applied to each patient. Nevertheless, as with any retrospective review, there were cases for whom the data were inconsistent or absent. Any records with missing critical data were excluded from review and analysis. The presence or absence of perianal symptoms was the one exception to this, and our previously described sensitivity analysis addressed this issue. In essence, 23 patients with absent information on perianal symptoms were all assumed to have perianal symptoms and, thus, moved to a less urgent referral scheme. This resulted in a lower calculated value for sensitivity. In reality, most of these patients were more likely not to have perianal symptoms, since these were not volunteered. We therefore submit that the tool’s true sensitivity lies closer to the higher estimate of 99.6% rather than the lower estimate of 91.5%.

The retrospective study design meant that all variables were collected from patient interviews, physical examinations and investigations done at or immediately following patient colonoscopy. The tool was designed to be applied at the initial point of care. This potential time delay of up to several weeks may have influenced the positivity of some of the tool criteria, and thus led to an overestimate of the tool’s sensitivity. The study was also therefore susceptible to recall bias, whereby patients, being aware of their diagnosis of CRC, over-recalled their initial symptomology when questioned. This effect would also potentially overestimate sensitivity. Our study sample was robust, with 281 confirmed cases of CRC to which to apply the tool in a mock fashion. However, it is conceivable that a larger sample would have yielded different results. Furthermore, this study was performed at a single tertiary centre, which could have implications on the generalizability of the findings when applied to remote or rural centers with varying access to endoscopy.

As only confirmed CRC cases were used for this analysis, other diagnostic properties of the tool such as specificity, negative predictive value and PPV could not be calculated. As such, one cannot interpret these data in isolation, as even a highly sensitive tool is of limited clinical use without an appropriate degree of specificity. While a tool with high sensitivity (in the absence of adequate specificity, PPV and negative predictive value) has identifiable limitations in application to clinical practice, the tool was intentionally developed by including only parameters above a robust median PPV threshold of 10%. PPV takes into account the sensitivity and specificity of the parameter as well as the prevalence of the underlying disease. The objective of this study was to ensure that the tool was inherently highly sensitive, thus, missing very few CRC cases with its application. Demonstrating the high sensitivity of the tool was deemed a crucial initial performance characteristic prior to proceeding with future prospective studies for comprehensive evaluation of the full diagnostic properties of the *CarePath-CRC* tool. Despite the high PPV of the individual components, we recognize the possibility that the overall tool’s PPV could be substantially lower when the individual criteria are used in combination. One of the most crucial properties of the tool requiring validation is its ability to exclude nonconcerning anorectal sources of bleeding, in an attempt to reduce the strain on urgent colonoscopy referral streams.

## CONCLUSION

Our *CarePath-CRC* tool shows robust sensitivity in the detection of CRC in patients initially presenting with rectal bleeding. Any evaluation of patients with suspected CRC should involve a thorough history which should include the presence or absence of perianal symptoms. The next phase in the validation of this tool will involve prospective evaluation and application of the tool at the point of primary care in order to determine its full operating characteristics.

## Funding

This study was supported by Canadian Association of Gastroenterology (CAG) Resident Research Award.

## Author Contributions

Conception and design: N.F., D.C., M.M., D.A., T.X. Analysis and interpretation of the data: all authors. Drafting of the article: N.F. Critical revision of the article for important intellectual content: all authors. Final approval of the article: all authors.
